# Impact of preS1 Evaluation in the Management of Chronic Hepatitis B Virus Infection

**DOI:** 10.3390/medicina60081334

**Published:** 2024-08-16

**Authors:** Yuka Hayashi, Kazuto Tajiri, Tatsuhiko Ozawa, Kiyohiko Angata, Takashi Sato, Akira Togayachi, Izuru Nagashima, Hiroki Shimizu, Aiko Murayama, Nozomu Muraishi, Hisashi Narimatsu, Ichiro Yasuda

**Affiliations:** 1 Third Department of Internal Medicine, Faculty of Medicine, Academic Assembly, University of Toyama, 2630 Sugitani, Toyama 930-0194, Japan; yukaberry0822@yahoo.co.jp (Y.H.);; 2 Department of Immunology, Faculty of Medicine, Academic Assembly, University of Toyama, 2630 Sugitani, Toyama 930-0194, Japan; 3 Center for Advanced Antibody Drug Development, University of Toyama, 2630 Sugitani, Toyama 930-0194, Japan; 4 Cellular and Molecular Biotechnology Research Institute, National Institute of Advanced Industrial Science and Technology, Tsukuba 305-8560, Japan; k.angata@aist.go.jp (K.A.); takashi-sato@aist.go.jp (T.S.); togayachi@soka.ac.jp (A.T.); izu.nagashima@aist.go.jp (I.N.); hiroki.shimizu@aist.go.jp (H.S.);

**Keywords:** HBsAg, preS1, HB surface protein, monoclonal antibody, HBV entry

## Abstract

*Background and Objectives*: The measurement of hepatitis B surface antigen (HBsAg) is essential for managing chronic hepatitis B virus infection (CHB). HBsAg consists of three different surface envelope proteins: large, middle, and small HB surface proteins. However, in clinical practice, it is not common to evaluate each of these HB surface proteins separately. *Materials and Methods*: In this study, we investigated preS1 expression using seven monoclonal antibodies (mAbs) in 68 CHB patients, as well as examining their antigenicity. *Results*: Although the seven mAbs had been derived from genotype (Gt) C, they could recognize preS1 with Gts A to D. The epitopes were concentrated within the aa33-47 region of preS1, and their antigenicity was significantly reduced by an aa45F substitution. We found that preS1 expression remained consistent regardless of HBsAg levels and different Gts in CHB patients, in contrast to what was observed in SHBs. *Conclusions*: These results suggest that the antigenic epitope is preserved among different Gts and that the expression pattern of preS1 is altered during CHB, highlighting its vital role in the HBV infection cycle. Our present results suggest preS1 is a promising therapeutic target in CHB.

## 1. Introduction

Chronic hepatitis B virus (HBV) infection is a serious problem, especially in Asia. About 250 million people suffer from chronic hepatitis B infection (CHB) [1], including about 1.1 million people in Japan [2]. CHB carries the risk of liver cirrhosis and hepatocellular carcinoma (HCC) [3]. In 2015, approximately 800,000 people died from CHB worldwide [4].

HBV is a DNA virus with an envelope protein including hepatitis B surface antigen (HBsAg). HBV is known to be differentiated into 10 genotypes (Gts) labeled from A to J [5]. The prevalence of HBV Gts varies geographically worldwide. Gt A is mainly found in Europe, and Gt D is prevalent in Africa, Europe, and India. In Japan, Gt C is the most prevalent (84.7%), followed by Gt B (12.2%) [6]. In clinical practice, HBsAg is a serological marker of HBV infection. Its primary function is to attach to the cell membrane of hepatocytes during the initial stages of infection [7]. The HBsAg envelope protein comprises three different surface proteins: large (LHBs), middle (MHBs), and small HBs (SHBs) [8,9]. SHBs includes the S domain, which is common to all three HB surface proteins. MHBs includes both the preS2 and S domains, while LHBs includes the preS1 (108 to 119 amino acids (aa), depending on the Gt), preS2, and S domains [10]. Among these, the preS1 sequence (aa2-47) plays an essential role in hepatocyte attachment [11,12,13,14]. Furthermore, preS1 has been shown to play an essential role also in replication and assembly in the life cycle of HBV [15].

The measurement of HBsAg is crucial in the management of CHB because HBsAg loss is considered a functional cure in CHB [16]. A previous study found that the development of HCC was markedly decreased in patients for whom HBsAg had disappeared with nucleos(t)ide analogue (NA) treatment, compared to those for whom it had not [17]. Sustained HBsAg loss after the completion of antiviral treatment is considered a functional cure of CHB and a therapeutic goal in CHB patients [18,19]. However, the measurement of HBsAg in clinical practice has mainly been conducted using polyclonal antibodies, primarily those targeting SHBs [20]. This approach enables the quantification of HBsAg load with high sensitivity. However, it poses challenges in qualitatively assessing the three HB surface proteins: SHBs, MHBs, and LHBs. Recent research has revealed distinct behaviors of MHBs and LHBs from those of SHBs before HBsAg loss. The quantification of preS1 has emerged as a potential marker of HBV viral status [21,22,23]. The role of a preS1 evaluation in the development of cirrhosis and HCC has also been demonstrated [24]. However, variation in the aa sequences in the preS1 domain has been shown according to the HBV Gt [25,26]. The quantification of preS1 specifically has not been fully integrated into daily clinical practice, partially due to the geographical variation of Gts, and further research is needed. To investigate HBsAg composition and the significance of preS1, we evaluated the component of HBsAg in CHB patients using the monoclonal antibodies (mAbs) for each HB surface protein and analyzed the significance of preS1 for future application to the clinical practice of CHB.

## 2. Materials and Methods

### 2.1. Preparation of Monoclonal Antibodies

PreS1 is an essential lesion for HBV and a potential therapeutic target. Therefore, we decided to acquire mAbs for preS1. We then obtained seven anti-preS1 mAbs using a cell-microarray system as previously described [27,28,29,30,31]. Briefly, mice (8-week-old, female, BALB/c) were immunized with LHBs protein with Gt C (Beacle Inc., Kyoto, Japan). All animal experiments were conducted with the approval of the Institutional Animal Committee at the National Institute of Advanced Industrial Science and Technology (AIST) and in compliance with the guidelines and regulations for animal care and use. After immunization and boost, spleen cells were applied to a microarray chip to detect single anti-preS1 antibody-secreting cells (ASCs) specific to the aa2-47 region in preS1 from Gt C. The ASCs were retrieved from the microchip, and antibody cDNAs for heavy- and light-chain variable fragments were amplified through single-cell reverse transcription-polymerase chain reaction (RT-PCR). To produce antibodies, we cotransfected Expi293F^TM^ cells (Thermo Fisher Scientific, Waltham, MA, USA) with both antibody heavy- and light-chain expression vectors. The antibodies were purified from the culture supernatant using a protein G column (Cytiva, Tokyo, Japan). Supplementary Table S1 shows a list of the mAbs used in the present study. Consequently, we obtained seven different anti-preS1 mAbs (L14, L28, L42, L43, L57, L58, L65). Additionally, we used an anti-preS2 antibody (F5) and an anti-SHBs antibody (HB1453) obtained through a similar method [29,32]. The origin of the anti-preS2 antibody was mice immunized with an *O*-glycosylated preS2 peptide of Gt C [32], and that of the anti-SHBs antibody was healthy volunteers vaccinated with yeast-derived non-infectious SHBs from Gt C [27,29]. L42, F5, and HB1453 were conjugated with alkaline phosphatase (ALP) using an ALP Labeling Kit-NH_2_ (DOJINDO, Kumamoto, Japan) following the manufacturer’s protocol. L28 was also conjugated with horseradish peroxidase (HRP) using an HRP Labeling Kit-NH_2_ (DOJINDO). Before using these conjugated mAbs (HRP-L28, ALP-L42, ALP-F5, ALP-HB1453), we confirmed their binding activity to the original antigen using an enzyme-linked immunosorbent assay (ELISA).

### 2.2. Binding Activity of Anti-preS1 mAbs in Different Genotypes

To examine the Gt specificity of anti-preS1 mAbs, we performed an ELISA. PreS1 peptides (aa 1-71) from Gt A, B, C, and D (Beacle) were diluted to a concentration of 1 μg/mL with PBS and added to a 96-well MaxiSorp-coated plate (Thermo Fisher Scientific). For blocking, a 3% bovine serum albumin (BSA) solution in phosphate buffered saline (PBS) was added to the plate. The anti-preS1 mAbs (L14, L28, L42, L43, L57, L58, L65) and control mouse monoclonal immunoglobulin (Ig) G1 (Abnova, Taipei, Taiwan) were diluted to various concentrations, namely, 1 μg/mL, 0.1 μg/mL, 0.01 μg/mL, and 0.001 μg/mL with 1% BSA in PBS, and added to the plate. Subsequently, ALP-conjugated secondary anti-mouse IgG (Sigma-Aldrich, St. Louis, MO, USA) was diluted to 1:1000 with 1% BSA in PBS and added to the plate. The absorbance value was measured at a wavelength of 405 nm using a microplate reader, Sunrise Rainbow (Tecan, Männedorf, Switzerland). All experiments were performed in duplicate.

### 2.3. Epitope Mapping

We designed several peptides for epitope mapping within the preS1 region. Since the anti-preS1 mAbs were generated using the preS1 peptide of amino acids (aa) 2-47 with Gt C, the epitope was considered to be within this region. We focused on the preS1 aa1-71 region according to the preS1 peptides experiments with different Gts, with the aa49-71 region designated as a negative control because it was considered a non-epitope region. To enable comprehensive mapping, we divided aa 1-71 into 15 aa long peptides with a 7 aa overlap, moving from the N-terminal to the C-terminal so that they can fully cover the epitope because several amino acids sequence is known to serve as an epitope of antibody. Consequently, we obtained 8 preS1 peptides (P1–P8). Biotin was added to the N-terminus of all peptides for easy binding to the microplate, and the purity of these peptides was confirmed to be ≥95%. In addition to these peptides, we designed peptides with single substituted amino acid sequences, namely, P5-G35K and P5-F45L, derived from P5. The sequences of these synthesized peptides are shown in Supplementary Table S2. All peptides were synthesized by GenScript Biotech Corporation, Piscataway, NJ, USA. We conducted epitope mapping of the seven anti-preS1 mAbs using the synthesized preS1 peptides. Each peptide was diluted to a concentration of 1 μg/mL with PBS. Additionally, LHBs recombinant protein (Beacle) was used as a standard and diluted to 1 μg/mL with PBS. Both the peptides and the LHBs recombinant protein were added to a streptavidin-coated 96-well plate (Nunc, Roskilde, Denmark) in duplicate for each antibody. After blocking with 3% BSA in PBS, the anti-preS1 mAbs (L14, L28, L42, L43, L57, L58, L65) and anti-SHBs mouse monoclonal IgG1 (Abnova), serving as negative controls, were diluted to 1 μg/mL with 1% BSA in PBS and added to the plate. The subsequent steps, including the addition of ALP-conjugated secondary Ab, the chromogenic reaction, and the measurement of absorbance, were carried out following the same procedure as described above.

### 2.4. Binding Activity of Anti-preS1 mAbs for an Amino Acid-Substituted Peptide

We performed an ELISA using anti-preS1 mAbs and peptides that had amino acid substitutions within the epitope. P5-G35K and P5-F45L peptides were diluted to a concentration of 1 μg/mL with PBS. Additionally, P5, serving as a standard, was also diluted to 1 μg/mL with PBS. The subsequent steps, including the blocking, addition of the anti-preS1 mAbs, addition of ALP-conjugated secondary Ab, chromogenic reaction, and measurement of absorbance, were carried out following the same procedure as described above.

### 2.5. Sample Collection

For CHB analyses, we included 64 chronic HBsAg-positive patients treated from March 2013 to February 2023 at Toyama University Hospital. All patients gave written informed consent. Serum HBsAg levels were measured using an Architect HBsAg QT assay (Abbott, North Chicago, IL, USA). The definitions of inactive carrier, chronic hepatitis, and cirrhosis and CHB treatment were in accordance with Japanese guidelines [33]. HBV-DNA levels were measured using a real-time PCR assay (COBAS TaqMan HBV Test; Roche, Tokyo, Japan). After isolating patient serum, it was transferred to microtubes and stored at −80 °C until use. In some cases, a switch from nucleos(t)ide analogs, such as entecavir (ETV), to tenofovir alafenamide fumarate (TAF) was performed according to the guidelines [33]. This study was conducted in accordance with the Declaration of Helsinki. The study protocol was approved by Toyama University Hospital Institutional Ethics Committee (R2019025).

### 2.6. Enzyme-Linked Immunosorbent Assay (ELISA)

An ELISA was used to measure the amount of each HB surface protein in the serum. MHBs was quantified using an HBsAgGi ELISA Kit (RCMG, Ibaraki, Japan) according to the manufacture’s protocol, while LHBs and SHBs quantification involved the following steps: A rabbit polyclonal antibody for HBsAg (Abnova) was diluted to 1 μg/mL with PBS and added to 96-well MaxiSorp-coated plates (Thermo Fisher Scientific). After blocking with 3% BSA, the patients’ serum was diluted to 1:100 with the dilution buffer. As a standard, SHBs protein (Aviva Systems Biology, San Diego, CA, USA) or LHBs protein (Beacle) was diluted to various concentrations (1 μg/mL, 500 ng/mL, 250 ng/mL, 125 ng/mL, 62.5 ng/mL, 31.25 ng/mL, 15.625 ng/mL) with 1% BSA in PBS as a dilution buffer. Thereafter, HRP-L28 or ALP-HB1453 was reacted with 1 μg/mL, and then those substrates were added to the plates. The absorbance value was measured using a microplate reader.

### 2.7. Western Blotting

To detect each HBs protein in the patients, we employed a Western blotting (WB) analysis. Furthermore, 7.5% Mini-PROTEAN TGX Precast Gels (Bio-Rad, Hercules, CA, USA) were used for a preS1 analysis because the weights of the LHBs (P39) were the most well separated; 12% Mini-PROTEAN TGX Gels (Bio-Rad) were used for an MHBs analysis and 4-20% Mini-PROTEAN TGX Gels (Bio-Rad) were used for an SHBs analysis for the best separation (MHBs:GP36, SHBs:P24). The serum samples of patients and those of healthy individuals used as negative controls were diluted 1:100 with PBS. LHBs protein (Beacle), *O*-glycosylated preS2 peptide [32], and SHBs protein (Aviva, San Diego, CA, USA) were used as positive controls. Each sample was applied to its respective gel, and proteins were separated via sodium dodecyl sulfate-polyacrylamide gel electrophoresis (SDS-PAGE) using PowerPac (Bio-Rad, Hercules, CA, USA). After SDS-PAGE, the resolved proteins were transferred to polyvinylidene difluoride membranes (Merck, Darmstadt, Germany). After blocking with a 4% BlockAce solution (Yukijirushi, Sapporo, Japan), ALP conjugated mAbs (ALP-L42, ALP-F5, and ALP-HB1453) were reacted. After treating with CDP-Star (Roche, Basel, Switzerland), an image Quant LAS4000 (GE HealthCare Technologies, Chicago, IL, USA) was used for visualizing immunoreactive bands.

### 2.8. Statistical Analyses

Patient characteristics were presented as the number or median and range. Experimental data were expressed as mean ± standard error (SE). Categorical variables were compared using the chi-square test or Fisher’s exact test, where applicable. Continuous variables were analyzed using the *t*-test or Mann–Whitney U test, where appropriate. Correlations between the total HBsAg and the load of each HB surface protein were evaluated using Spearman’s rank test to determine correlation coefficients. The absorbance of each HB surface protein after TAF switching divided by the absorbance just before switching was defined as the “change ratio”. The change ratios after TAF switching were expressed as mean ± SE. A *p*-value < 0.05 was deemed statistically significant. All statistical analyses were performed using JMP Pro17.0 software (SAS Institute, Cary, NC, USA).

## 3. Results

### 3.1. Binding Activity of Anti-preS1 mAbs against preS1 Peptides with Genotypes A–D

**Figure 1:**
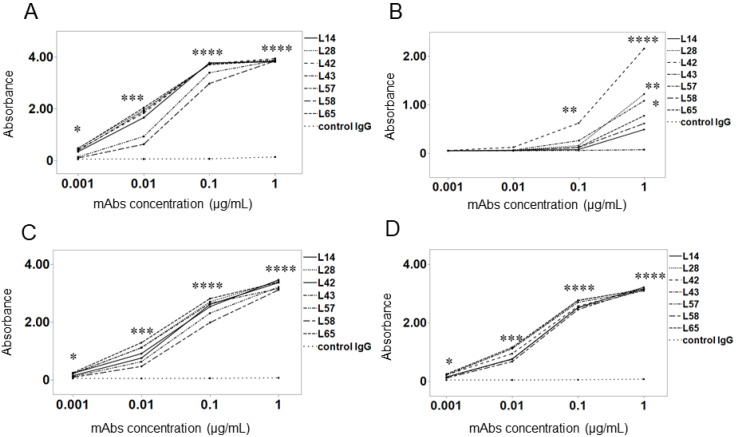
Binding activity of anti-preS1 mAbs to preS1 peptides with genotypes A-D. *Y*-axis: Absorbance at 405 nm. *X*-axis: Concentration of mAbs (μg/mL). (**A**) Gt A. (**B**) Gt B. (**C**) Gt C. (**D**) Gt D. Data represent mean absorbance in duplicate. Control IgG serves as the negative control for anti-preS1 mAbs. * *p* < 0.05, ** *p* < 0.01, *** *p* < 0.001, **** *p* < 0.0001 using Student’s *t*-test. All statistical differences were compared between the mAbs and control IgG.

We examined whether anti-preS1 mAbs could bind to various Gts of the preS1 peptide. All seven mAbs exhibited significantly stronger binding activity to the preS1 peptides from Gts A to D than the control (Figure 1A–D). Importantly, all anti-preS1 mAbs were capable of binding to the Gt A–D peptide despite being generated from the preS1 peptide (aa 2-47) with Gt C. There was no significant difference in binding activity among the mAbs. Regarding Gt B, binding activity was slightly decreased compared with other Gts, but remained stronger than in the control. In particular, L42, L28, and L43 showed significantly stronger binding than the control at a concentration of 1 μg/mL (Figure 1B).

### 3.2. Epitope Analyses of Anti-preS1 mAbs

**Figure 2:**
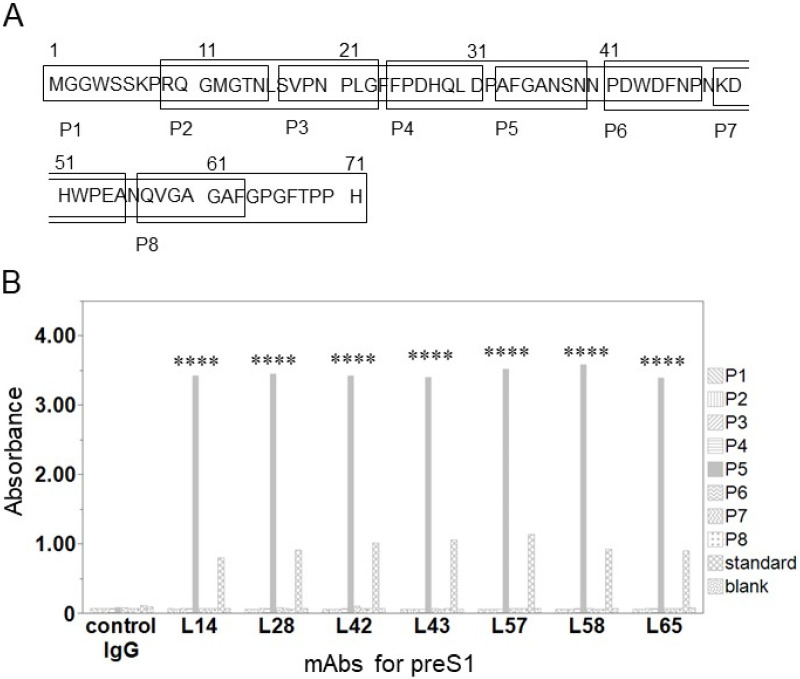
(**A**) N-terminal amino acid sequence of preS1 (Gt C) and sequences of synthesized peptides P1–P8. P7 and P8 were used as negative controls. (**B**) ELISA to assess the binding activity of P1–P8 and anti-preS1 mAbs. Mean values of duplicate absorbance are shown. Standard: recombinant LHBs protein; blank: PBS; control IgG: negative control for anti-preS1 mAbs. **** *p* < 0.0001 using Student’s *t*-test.

We evaluated the epitopes recognized by the anti-preS1 mAbs. The epitope mapping of the synthesized peptides, P1-8, is shown in Figure 2A. When analyzing the binding activity of the anti-preS1 mAbs with the P1–P8 peptides, we found that all seven mAbs designed for preS1 aa 2-47 exhibited strong binding to P5 (aa33-47) (Figure 2B). The binding activity for P5 was stronger than that for the LHBs protein, and none of the mAbs bound to the other synthesized peptides.

### 3.3. Identification of Single Amino Acid Causing Weak Binding between Anti-preS1 mAbs and preS1 of Gt B

**Figure 3:**
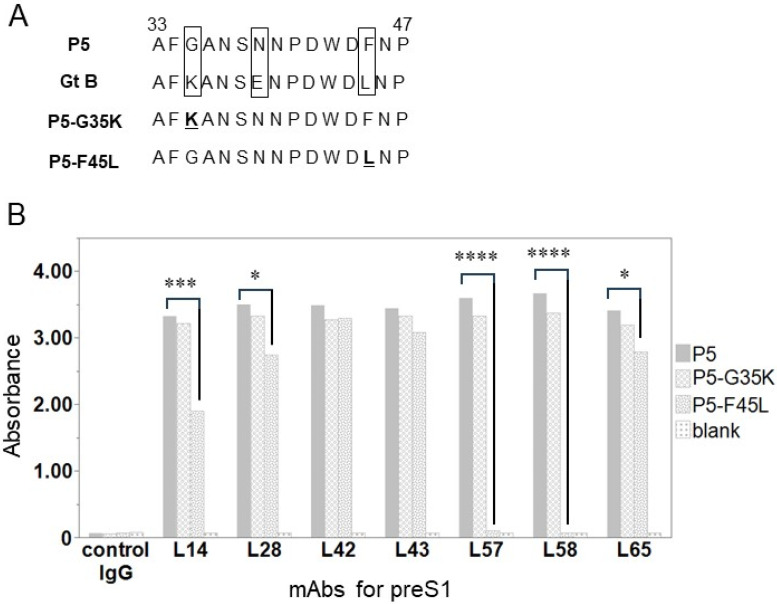
Analysis of binding activity between substituted amino acid peptide and anti-preS1 mAbs. (**A**) The sequence of P5, the sequence with Gt B corresponding to P5, the substituted peptide of P5-G35K and P5-F45L. Boxes at 35, 39 and 45 aa position mean the difference between P5 (Gt C) and Gt B, Underlines of P5-G35K and P5-F45L mean substituted amino acid. (**B**) Analysis of binding activity between substituted amino acid peptides and anti-preS1 mAbs. Mean values of duplicate absorbance are shown. Standard: recombinant LHBs protein; blank: PBS; control IgG; mAb for SHBs. * *p* < 0.05, *** *p* < 0.001, **** *p* < 0.0001 using Student’s *t*-test.

The binding activity between the preS1 of Gt B and anti-preS1 mAbs was slightly decreased compared with other Gts (Figure 1B), and the epitope of the mAbs was found within P5 (Figure 2). Next, we assessed the binding activity of the anti-preS1 mAbs when single amino acid substitutions were introduced in the P5 peptide. By comparing the sequence of P5 with that of Gt B, different amino acid sequences were found (Figure 3A). We designed the P5-G35K peptide, in which G35 was substituted with K35, and the P5-F45L peptide, in which F45 was replaced with L45 (Figure 3A, Supplementary Table S2). We found that all anti-preS1 mAbs bound to P5-G35K similarly to P5. However, L42 and L43 displayed strong binding to P5-F45L, similar to their binding to P5, while the binding activity of L28 and L65 decreased. The binding activity of L14 decreased even more, and that of L57 and L58 disappeared completely (Figure 3B).

### 3.4. Patient Characteristics

Next, we evaluated the expression of preS1 in a clinical setting. Table 1 presents the baseline patient characteristics evaluated in the present study. The median age was 66 years (range: 30–87), and 36 were male. Most patients exhibited a chronic hepatitis status, followed by inactive carrier and cirrhosis statuses. Among them, 25 had HBsAg levels < 100 IU/mL, 18 had levels between 100 and 1000 IU/mL, and 21 had levels exceeding 1000 IU/mL. Regarding Gt, Gt C was the most common, followed by Gt B. HBV-DNA was undetectable in about half of the patients. Among those in whom HBV-DNA was detectable, the median level was <1.0 Log IU/mL. Most patients were HBeAg-negative (49/61, 80.3%). Among the patients, 48 received antiviral therapy, while 16 were treatment-naive. The primary antiviral therapy consisted of NAs, with 31 patients receiving ETV, 8 receiving TAF, and 1 receiving tenofovir disoproxil fumarate (TDF) alone.

### 3.5. Correlation between Each HB Surface Protein and Total HBsAg

**Figure 4:**
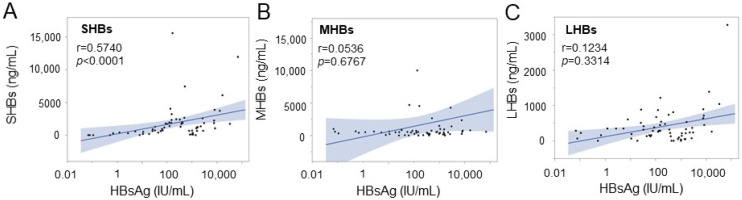
Correlations between HBsAg load (IU/mL) (*X*-axis) and concentration of SHBs (**A**), MHBs (**B**), and LHBs (**C**) (ng/mL, *Y*-axis). Correlation was determined using Spearman’s rank correlation coefficient (r).

**Figure 5:**
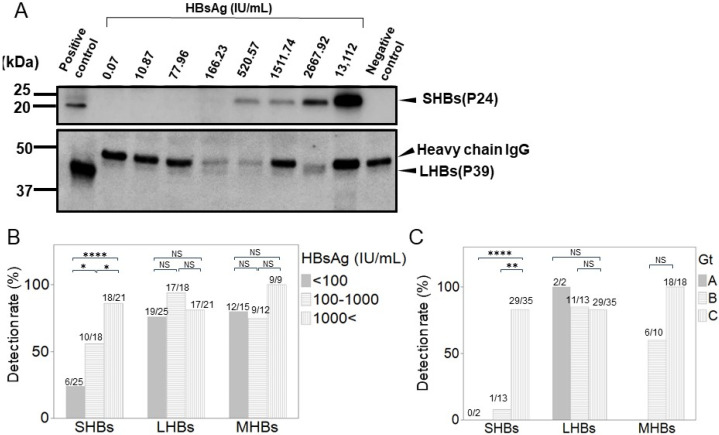
(**A**) Representative WB data with respect to HBsAg levels. Numbers on top of the column represent the HBsAg level of the sample. The positions of SHBs and LHBs bands are indicated on the right. In the LHBs data, non-specific bands corresponding to the heavy chain of IgG (IgG-HC) were found. (**B**) Detection rates of SHBs, MHBs, and LHBs in patient serum based on HBsAg levels (HBsAg < 100 IU/mL, 100–1000 IU/mL, and >1000 IU/mL). The number on top of each bar graph represents the number of patients with positive/analyzed data. (**C**) Detection rates of SHBs, MHBs, and LHBs in patient serum for Gts A, B, and C. NS: not significant; * *p* < 0.05, ** *p* < 0.01, **** *p* < 0.0001 using chi-square test.

We performed an ELISA to quantify the levels of SHBs, MHBs, and LHBs in the CHB patients’ serum and compared the results with the total HBsAg load. SHBs showed a significant positive correlation with the total HBsAg load (r = 0.5740, *p* < 0.0001, Figure 4A). However, MHBs and LHBs had no significant correlation with the total HBsAg load (r = 0.0536, *p* = 0.6767; r = 0.1234, *p* = 0.3314, respectively) (Figure 4B,C). We performed an ELISA to quantify the levels of SHBs, MHBs, and LHBs in the CHB patients’ serum and compared the results with the total HBsAg load. SHBs showed a significant positive correlation with the total HBsAg load (r = 0.5740, *p* < 0.0001, Figure 5A). However, MHBs and LHBs had no significant correlation with the total HBsAg load (r = 0.0536, *p* = 0.6767; r = 0.1234, *p* = 0.3314, respectively) (Figure 4B,C).

### 3.6. Detection of Each HB Surface Protein Using WB in Various CHB Patients

We then performed a WB analysis to separately detect each HB surface protein in the serum of the CHB patients. The detection of SHBs depended on the HBsAg load, and they were significantly decreased in patients with HBsAg < 100 IU/mL, consistent with the results of ELISA (Figure 5A,B). However, LHBs could be consistently detected, regardless of a decrease in HBsAg (Figure 5A). Furthermore, MHBs was detected consistently, regardless of a decrease in HBsAg (Supplementary Figure S1). Subsequently, the patients were categorized based on their serum HBsAg load (HBsAg < 100 IU/mL, 100–1000 IU/mL, and >1000 IU/mL), and the detection rates of the HB surface proteins were then compared. LHBs and MHBs were highly detectable independent of HBsAg load, with no significant differences. However, compared with that of LHBs or MHBs, the detection rate of SHBs significantly decreased with a reduction in the HBsAg load (Figure 5B). Next, we compared the detection rates among HBV Gts A, B, and C. SHBs could not be detected in Gt A but could be detected in most patients with Gt C (Figure 5C). In Gt B, SHBs was rarely detected, except for in one patient with an HBsAg level of 8440 IU/mL. In contrast, LHBs could be detected in most patients with Gts A, B, and C (Figure 5C).

### 3.7. Each HB Surface Protein Levels According to HBeAg Status and HBV-DNA

**Figure 6:**
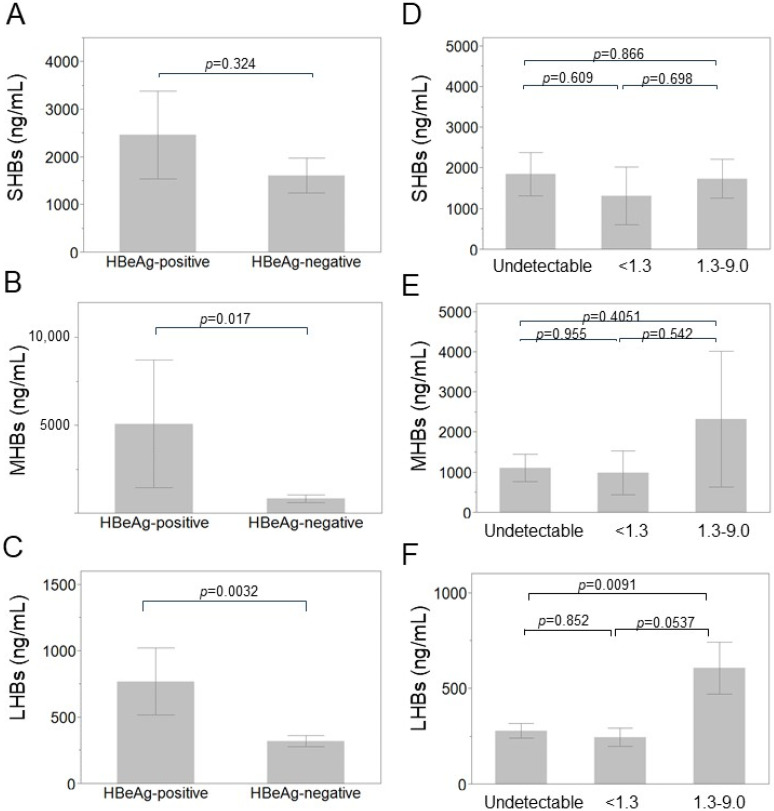
Each HB surface protein according to HBeAg status (**A**–**C**) and HBV-DNA (**D**–**F**). (**A**) SHBs in patients with HBeAg-positive and HBeAg-negative. (**B**) MHBs in patients with HBeAg-positive and HBeAg-negative. (**C**) LHBs in patients with HBeAg-positive and HBeAg-negative. (**D**) SHBs in patients with HBV-DNA undetectable, <1.3 LogIU/mL and 1.3–9.0 LogIU/mL. (**E**) MHBs in patients with HBV-DNA undetectable, <1.3 LogIU/mL and 1.3–9.0 LogIU/mL. (**F**) LHBs in patients with HBV-DNA undetectable, <1.3 LogIU/mL and 1.3–9.0 LogIU/mL. Statistical difference was compared using Student’s *t*-test.

We then analyzed the relation between HB surface proteins and HBV-DNA levels or hepatitis B envelope antigen (HBeAg) status. When we compared the levels of LHBs, MHBs, and SHBs determined by ELISA according to HBeAg status, SHBs showed no significant difference between the HBeAg-positive and -negative patients (*p* = 0.324, Figure 6A). However, MHBs and LHBs showed a significant increase in HBeAg-positive patients (*p* = 0.017 and 0.0032, respectively, Figure 6B,C). Regarding HBV-DNA, SHBs levels showed no significant difference according to HBV-DNA levels, but LHBs showed a significant increase in the patients in the HBV-DNA-positive group (*p* = 0.0091, Figure 6D–F). SHBs showed a good correlation with HBsAg levels independent of HBeAg status or HBV-DNA positivity (left panels in Supplementary Figure S2). However, LHBs showed a correlation with HBsAg level only in patients with HBeAg-positive or HBV-DNA detectable, but not in those with HBeAg-negative or HBV-DNA undetectable (right panels in Supplementary Figure S2). MHBs showed no correlation with HBsAg levels irrespective of HBeAg or HBV-DNA status in the present study (middle panels in Supplementary Figure S2).

### 3.8. Changes in HB Surface Proteins During Switch from ETV to TAF

To investigate the clinical significance of preS1, we conducted a preliminary investigation into the changes in each HB surface protein in 10 CHB patients during the switch from ETV to TAF using an ELISA. Their clinical characteristics are shown in Supplementary Table S3. In this preliminary analyses, Gt C was prevalent, and the median HBsAg level before the switch was 237.7 IU/mL. The HBsAg level remained unchanged during the 12 months after switching to TAF (comparing 0 months with 6 months (0–6): *p* = 0.3393; comparing 0 months with 12 months (0–12): *p* = 0.2599) (Supplementary Figure S3A). SHBs and MHBs also remained unaltered during the 12 months after switching (0–6, *p* = 0.6411, 0–12, *p* = 0.9110; 0–6, *p* = 0.5388, 0–12, *p* = 0.6424, respectively) (Supplementary Figure S3B,C). However, a significant increase was observed in only LHBs after 6 months and 12 months (0–6, *p* = 0.0248, 0–12, *p* = 0.0057) (Supplementary Figure S3D).

## 4. Discussion

In this study, we identified the epitope of preS1 mAbs, which was concentrated in the aa33-47 region of preS1, and found that aa45F plays a crucial role as an antigenic epitope. The amino acid sequence of this region is conserved among different HBV Gts, and the antigenic epitope was preserved across Gts. This region has been shown to play an essential role in HBV entry into hepatocytes through its interaction with the sodium taurocholate co-transporting polypeptide [12,34,35,36]. These findings suggest novel therapeutic options such as vaccination or target therapies with this epitope are expected to be explored. Furthermore, we demonstrated that the expression of preS1 is maintained independently of HBsAg load and that it differs from the expression of SHBs in CHB patients. Although the preS1 expression is largely found in patients with HBV virions, it is preserved in those with decreased HBsAg. The results suggest the preS1 is preserved throughout HBV infection and across various Gts due to its vital roles in HBV.

In present study, we found that preS1 expression was consistently maintained across different HBV Gts, as evidenced in CHB patients with Gts A, B, and C. This was supported by our experiments with preS1 peptides from Gts A to D, although the binding activities of anti-preS1 mAbs were relatively weaker for Gt B. Differences in amino acid sequences in preS1 corresponding to different Gts were reported [15]. A previous report also showed that LHBs levels were correlated with HBsAg levels independent of Gt [37]. However, in our study, we found that the sequence of the Gt C peptide at aa33-47 (P5, AFGANSNNPDWDFNP) differed from that of the Gt B peptide (AFKANSENPDWDLNP) by three amino acid substitutions (G35K, N39E, and F45L). We hypothesize that G35K or F45L substitutions might be responsible for the decreased binding activity of the Gt B peptide. A significant decrease in binding activity was observed with the single amino acid-substituted peptide P5-F45L, suggesting that F45 may play a crucial role in the antigenicity of preS1. This antigenic epitope was almost preserved across Gts A to D, suggesting the clinical significance for therapeutic application of this epitope.

Regarding with preS1, previous studies have reported the significance of aa21-47 within preS1 as a highly antigenic region [15,38]. Various reports have indicated that anti-preS1 antibodies can recognize specific sequences within this region, with one study suggesting aa25-38 as the target [39], and others have identified epitopes at aa 37-45 [40] and aa38-47 [37]. Our findings are consistent with the findings of these studies. PreS1 is known to facilitate the attachment of HBV viral particles to host hepatocytes. A prominent binding site for hepatocytes was identified within the aa21-47 region [12]. While this region is indispensable for the survival of HBV, it is highly immunogenic, serving as a key interface between the virus and the host immune system. Consequently, it may be a promising diagnostic and therapeutic target. Recent studies have explored the potential clinical applications of measuring preS1 [15,23,24,37,39,41,42]. A recent study showed that preS1 decrease is a predictor of HBsAg clearance [43]. Furthermore, preS1 expression remained until the end of HBsAg clearance [43], although preS1 expression was found with low-frequency on HBV virions as mentioned above [44,45]. A recent review also indicated that HBsAg isoform quantitation can become a useful non-invasive biomarker for assessing CHB [43]. As for treatment with preS1, vaccination with preS1-containing SVPs showed superior immunogenic and protective effects than conventional vaccine with SVPs with SHBs only [46,47,48]. Furthermore, inhibition of HBV secretion through preS1 interaction was also shown [49].

In clinical practice, measurement of HBsAg with SHBs, rather than LHBs or MHBs, has mainly been performed. SHBs is a major component in viral particles, present in both infectious viral particles and non-infectious subviral particles (SVPs) [44]. The expression of preS1 has been reported to be low on HBV virions (<10%) [44,45] and even lower on SVPs [45]. Our data also indicate that preS1 is expressed on HBV virions. A recent study suggested that the expression of LHBs and MHBs in patients with an inactive carrier status is lower than in those with CHB [22]. This implies that there might be functional alterations in LHBs and MHBs during HBV infection, especially after HBe seroconversion or HBV virions clearance in the serum of CHB patients. Therefore, it is essential to carefully consider the evaluation of LHBs or MHBs. In this study, the assessment of MHBs was performed using a mAb for *O*-glycosylated preS2. The contribution of glycosylation in LHBs remains uncertain in the present study. Complex glycosylation has been implicated in HBV infection and its role in the CHB cycle [32,50,51]. Further investigations regarding glycosylic modifications of HBV and their relationship with HBV infection status are required. In addition, unknown underlying mechanisms in preS1, preS2, or the S domain produced as SVPs during CHB might be found. Further research is needed to assess its applicability in daily clinical practice.

A reduction in HBsAg is a desirable goal in the management of CHB. However, achieving this is challenging with NAs [52]. Tenofovir has shown potential to induce interferon λ more effectively than drugs such as ETV acting as nucleotide analogs [53]. As a result, the switch from ETV to TAF has been explored, particularly in Japan. TDF/TAF was reported to decrease HBsAg levels compared with ETV [54,55]; however, these results are controversial [56,57]. In this study, we observed a different pattern in LHBs levels after switching from ETV to TAF, although it was preliminary. This suggests that TAF switching may exert some pressure on HBV. A mechanism by which HBV tries to survive under anti-viral stress might have occurred. Further investigations are required to elucidate the precise mechanisms underlying the expression pattern alteration of LHBs after transitioning to TAF and to assess its long-term implications.

This study has the following limitations: It included a limited number of patients. Data concerning different genotypes, HBV status, and TAF switching were scarce. Furthermore, other essential data, such as circulating HBV-RNA, were not evaluated in daily clinical practice. In the present study, we investigated changes in each HB surface protein in TAF-switching patients because such NA switching is recommended in Japanese clinical guidelines and has been investigated in terms of its efficacy and tolerability [33]. Investigating changes in each HB surface protein in patients before and after NA stopping might be more valuable. While we identified the importance of the aa33-47 region, particularly F at aa45, as a crucial epitope in preS1, we did not conduct functional assays to elucidate its precise role. Investigating its function could have valuable therapeutic implications. Furthermore, we investigated the antigenicity of preS1 mAbs for only Gts A, B, C and D. PreS1 peptides with Gts E to J were not available in present study. Future investigations are desired. The expression of glycosylated SHBs or LHBs was uncertain in the present study. The antigenicity of mAbs for glycosylated LHBs was not evaluated; thus, further research on this is needed in future studies. An assessment of preS1 expression in CHB patients was conducted using WB and an ELISA, which may be unsuitable for routine use due to effort and sensitivity. More effort is required to detect preS1 in patients with very low HBsAg. A simple and sensitive quantitative method capable of covering other Gts, such as Gts E to J, is desired for clinical application.

## 5. Conclusions

We report that the expression of preS1 remains consistent in CHB patients regardless of HBsAg load and Gt. The aa 33-47 region in preS1, especially amino acid F at position 45, is highly antigenic, but it is preserved among different Gts. This region plays a crucial role in the CHB life cycle, suggesting its potential as a diagnostic and therapeutic target for CHB patients. Further research is needed for its application in clinical practice.

## Figures and Tables

**Table 1:** Clinical characteristics of patients.

Variables	Number or Median (Range)
Patients, *n*	64
Age (years), median (range)	66 (30–87)
Sex, *n*	
Male	36
Female	28
CHB status, *n*	
Chronic hepatitis	28
Cirrhosis	11
Inactive carrier	25
HBsAg (IU/mL), *n*	
<100	25
100–1000	18
1000<	21
Genotype, *n*	
A	2
B	13
C	35
Not determined	14
HBV-DNA (Log IU/mL), *n*	
Undetectable	31
Detectable (<1.3)	8
Detectable (range: 1.3–9.0)	25
HBeAg, *n*	
Positive	12
Negative	49
Not examined	3
Antiviral therapy, *n*	
ETV	31
TAF	8
TDF	1
LAM	1
Combined regimen	7
Treatment-naïve	16

CHB, chronic hepatitis B infection; ETV, entecavir; TAF, tenofovir alafenamide fumarate; TDF, tenofovir disoproxil fumarate; LAM, lamivudine. Combined regimens included LAM, adefovir pivoxil, ETV, pegylated interferon alfa-2a, and TDF.

## Data Availability

The data shown in this study are available from the corresponding author upon reasonable request.

## Supplementary Materials

The following supporting information can be downloaded at https://www.mdpi.com/article/10.3390/medicina60081334/s1, Table S1, Characteristics of mAbs used in present study; Table S2, Amino acid sequences of synthesized preS1 peptides; Table S3, Characteristics of patients switching from ETV to TAF; Figure S1, Representative data of western blotting for MHBs; Figure S2, Correlation between each HB surface protein and HBsAg according to HBeAg status or HBV-DNA positivity; Figure S3, Changes in HBsAg and each HB surface protein after switching from ETV to TAF.
