# Supplementary material for: Impact of preS1 Evaluation in the Management of Chronic Hepatitis B Virus Infection
Source: Medicina (Kaunas). 2024 Aug 16;60(8):1334. doi: 10.3390/medicina60081334 (PMC11356368; doi:10.3390/medicina60081334)
Supplement: Supplementary file 1 [file medicina-60-01334-s001.zip › medicina-3139000-supplementary.pdf]

Supplementary Table S1. Characteristics of mAbs for each HBs protein

mAbs	Antigen	Origin
HB1453	Yeast-derived S-HBs protein (Gt C)	Human
F5	O-glycosylated preS2 peptide aa17-48 (Gt C)	Mouse
L14, L28, L42, L43, L57, L58, L65	preS1 peptide aa 2-47 (Gt C)	Mouse

mAbs: monoclonal antibodies, Gt: Genotype

Supplementary Table S2. Amino acid sequences of synthesized preS1 peptides

Peptides	Amino acid	Sequence
P1	1-15	MGGWSSKPRQGMGTN
P2	9-23	RQGMGTNLSVPNPLG
P3	17-31	SVPNPLGFFPDHQLD
P4	25-39	FPDHQLDPAFGANSN
P5	33-47	AFGANSNNPDWDFNP
P6	41-55	PDWDFNPNKDHWPEA
P7	49-63	KDHWPEANQVGAGAF
P8	57-71	QVGAGAFGPGFTPPH
P5-G35K	33-47	AFKANSNNPDWDFNP
P5-F45L	33-47	AFGANSNNPDWDLNP

Supplementary Table S3. Characteristics of the patients switched from ETV to TAF

No	Age	Sex	CHB status	HBsAg (IU/mL)	Genotype
1	69	F	Chronic hepatitis	0.52	Not determined
2	76	M	Chronic hepatitis	0.69	Not determined
3	77	F	Cirrhosis	32.88	C
4	70	F	Cirrhosis	33.55	C
5	55	M	Cirrhosis	56.16	C
6	70	M	Cirrhosis	77.96	C
7	61	M	Chronic hepatitis	137.13	B
8	64	F	Chronic hepatitis	149.69	Not determined
9	51	F	Inactive carrier	377.12	B
10	74	F	Chronic hepatitis	1511.74	C

Supplementary Figure S1. WB of MHBs

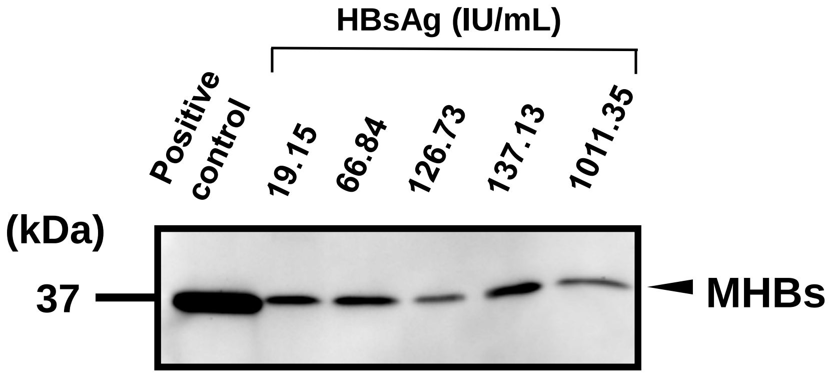

Supplementary Figure S2. Correlation between each HB surface protein and HBsAg levels according to HBeAg status or HBV-DNA positivity.

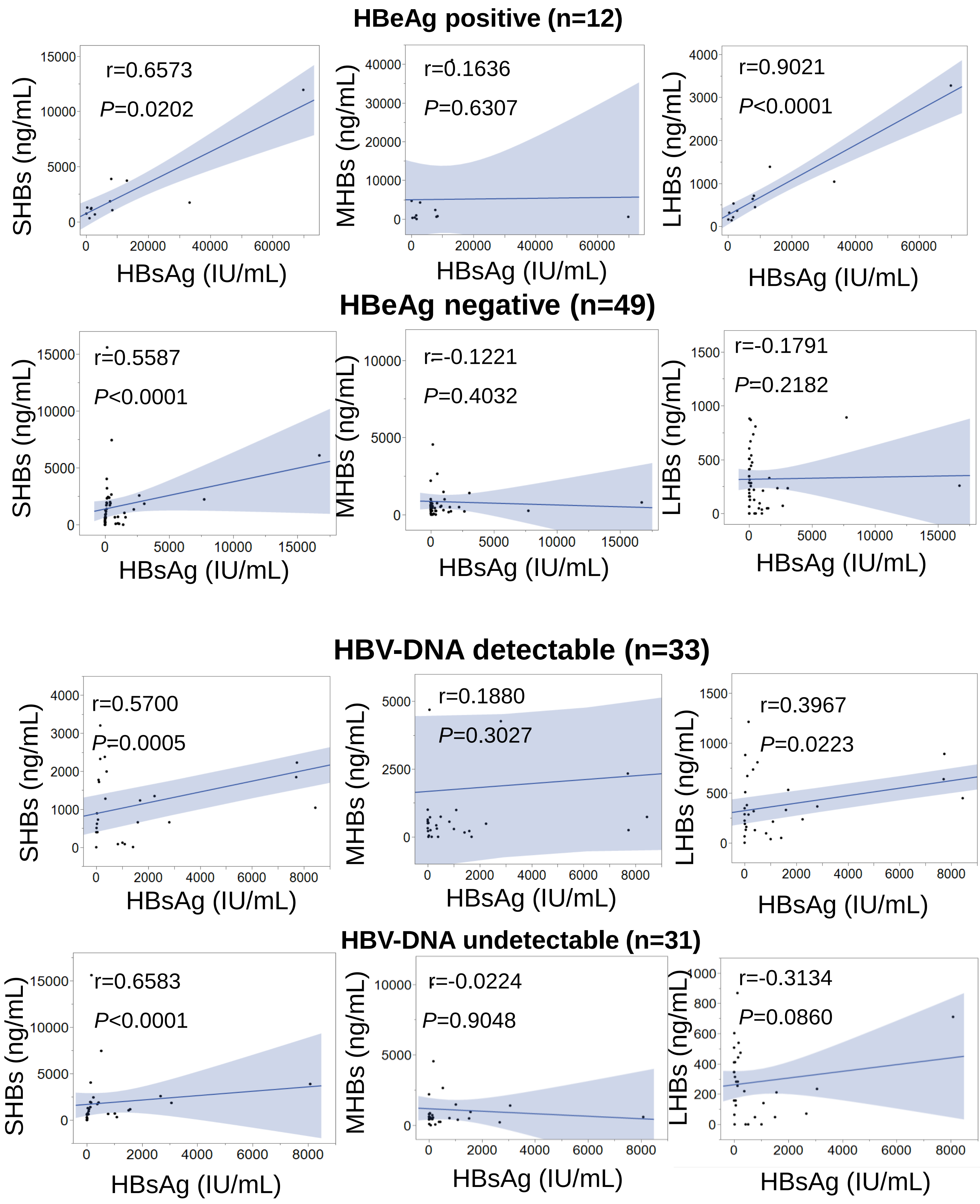

Supplementary Figure S3. Changes in HBsAg and each HB surface protein after switching from ETV to TAF

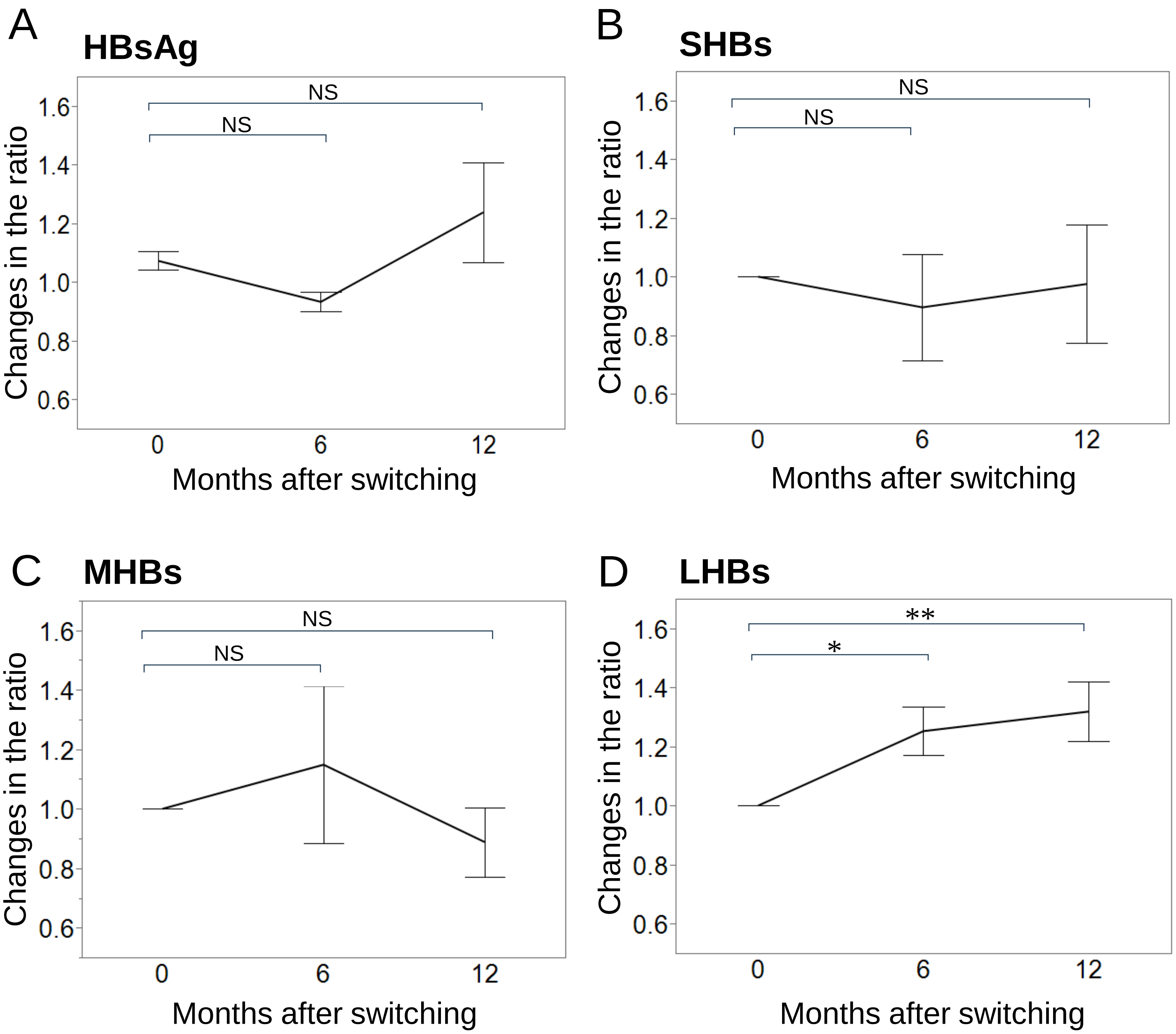

Y-axis: changes in the ratio (mean ± SE) as compared to baseline. The value at the start of switching (X-axis 0) was set to 1. X-axis: months after switching from ETV to TAF. **A)** HBsAg, **B)** SHBs, **C)** MHBs, **D)** LHBs. NS: not significant; * P<0.05, ** P<0.01 using Student's t-test.
